# Arnold-Chiari malformation and significant lumbar disc prolapse in pregnancy: A case report and literature review

**DOI:** 10.1016/j.crwh.2021.e00337

**Published:** 2021-06-19

**Authors:** Albert Adu Opoku, Gisha Varghese Mathew, A. Thode, K. Noureddine

**Affiliations:** a Al Wakra Hospital, Hamad Medical Corporation, P. O Box 82228, Al Wakra, Qatar; b Weill Cornell Medicine, P. O Box 24144, Doha, Qatar

**Keywords:** Arnold-Chiari malformation, Case report, Lumbar disc prolapse, Pregnancy, Regional anesthesia

## Abstract

A 30-year-old woman (gravida 3, para 1 + 1), with a previous uncomplicated pregnancy and vaginal delivery, was diagnosed with both type 1 Arnold-Chiari malformation and symptomatic multi-level lumbar disc prolapse in her inter-pregnancy period. During this index pregnancy, she experienced progressively worsening occipital headaches radiating to both arms, severe low back pain radiating to both legs and weakness in both legs. She had no urinary or bowel symptoms. She was successfully managed through pregnancy by a multidisciplinary team that included obstetricians, orthopedic and neurosurgeons, obstetric anesthetists and physiotherapists. She had an uncomplicated cesarean delivery under spinal anesthesia. As far as we can tell, this is the first report of both conditions in a pregnant woman.

## Highlights

• Arnold-Chiari malformation in pregnancy is rare.
• Patient care is best individualized and managed by a multidisciplinary team.
• Regional and general anesthesia can have patient specific challenges for women with Arnold Chiari malformation in pregnancy.
• Women with disc prolapse in pregnancy need urgent MRI and neurosurgical review for new or worsening neurological symptoms.
• Spinal decompression surgery can be performed at all stages of pregnancy when indicated.

## 1 Introduction

Pregnancy in women with Arnold-Chiari malformations (ACM) present unique challenges to obstetricians and anesthetists in management and delivery. Pregnancy itself, uterine contractions and maternal effort during vaginal delivery could increase intracranial pressure, aggravate maternal symptoms and worsen the condition. Anesthesia for labor analgesia or obstetric surgery is a matter of debate as general, epidural or spinal anesthesia can all have consequences [1,2].

Low back pain is common in pregnancy; on rare occasions it may be due to lumbar disc prolapse. The increasing size of the gravid uterus as well as the physiological changes in pregnancy are thought to progressively worsen this condition [[3], [4], [5]]. Effective regional anesthesia for labor analgesia and operative delivery can both be a challenge in women with significant lumbar disc prolapse.

This patient had both conditions and was successfully managed though pregnancy and cesarean delivery with a multidisciplinary team approach.

## 2 Case Presentation

A 30-year-old woman (G3 P1 + 1) booked for antenatal care at seven weeks of gestation. She had had an uncomplicated pregnancy with spontaneous vaginal delivery two and a half years previously, with epidural analgesia, as well as a prior miscarriage.

**Fig. 1:**
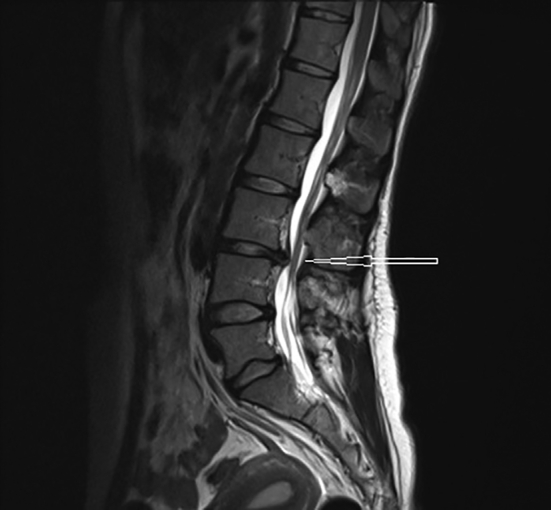
Magnetic resonance scan of the lumbar spine at L3/L4.

**Fig. 2:**
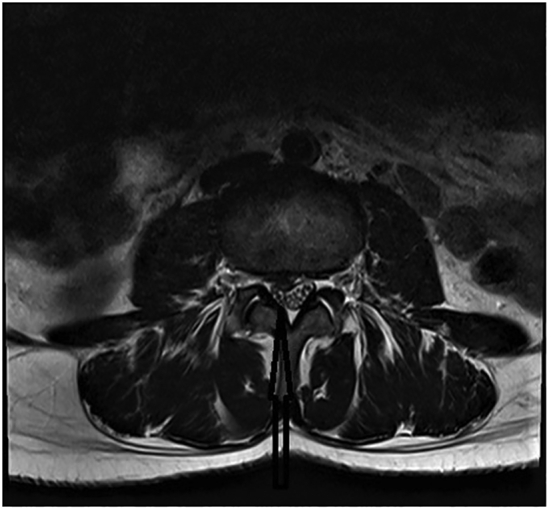
Magnetic resonance scan of the lumbar spine at L3/L4 (transverse view).

**Fig. 3:**
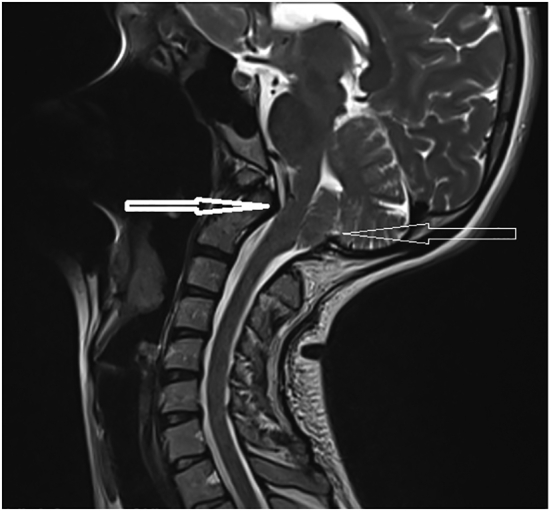
Odontoid (bold arrow), tonsillar herniation (arrow).

A year after her first delivery, she presented to the emergency department with severe back pain, worse on the right side. An X-ray of her spine showed instability of the third to fifth lumbar vertebrae, with widening of the disc spaces. She was referred to the orthopedic surgeons and had progressive worsening of her back pain and new-onset weakness in her right leg over the course of one year. Magnetic resonance imaging (MRI) of her lumbar spine revealed multi-level lumbar disc prolapse involving the third lumbar vertebra (L3) to the first sacral vertebra (S1), worse at L3/L4 (see Fig. 1, Fig. 2). Spinal surgery was discussed, but the patient declined at the time and was managed conservatively. She was also diagnosed with type 1 Arnold-Chiari malformation (Fig. 3) six months prior to her index pregnancy, following complaints of neck pain radiating to both arms, with tingling, numbness and shooting pains, and was under the care of neurosurgeons. Her last MRI scan was a month before pregnancy and did not show any significant progression in her disease.

Her main symptoms at booking were occipital headaches radiating to both arms, severe low back pain radiating to both legs, worse on the right, and weakness in both legs. She did not have any bowel or bladder dysfunction. She was managed mainly with paracetamol. She continued with her physical therapy remotely due to the COVID-19 pandemic and could not access hydrotherapy, which had previously been helpful.

Her pregnancy was otherwise uncomplicated, with regular follow-up in the consultant antenatal clinic. She had anesthetic consultations at 30 weeks of gestation and again at 36 weeks to plan her anesthetic management. She also had both neurosurgery and orthopedic consultations at 14 and 30 weeks of gestation.

Both her neurosurgeon and orthopedic surgeon recommended delivery by cesarean section (CS) under regional anesthesia at a multidisciplinary discussion between all consulting physicians, due to the potential risks of aggravating her disc prolapse by an attempt at vaginal delivery. This was also the preferred option of the patient. General anesthesia (GA) presented potential dangers, as head extensions and raised intracranial pressures could aggravate her neurological symptoms. She had a CS planned for 38 weeks of gestation, but she presented the night before her scheduled CS with spontaneous rupture of membranes and mild contractions. She had an uncomplicated emergency CS under spinal anesthesia done by the head of obstetric anesthesia, using ultrasound guidance.

Her immediate post-operative period was uncomplicated; there were no neurological symptoms nor worsening of her existing symptoms. She was discharged home on the second day post-delivery. No new developments were reported in follow-up calls after two weeks and one month.

## 3 Discussion

The Arnold-Chiari malformation was first identified in 1883 by Cleland. It is characterized by the prolapse of hindbrain structures below the level of the foramen magnum. It can be associated with skeletal abnormalities and neurologic dysfunction. Chiari divided it into four classes. Type 1, with displacement of cerebellar tonsils below the foramen magnum, is usually diagnosed in adults. Type 2, with displacement of the cerebellar vermis, brainstem, and fourth ventricle through the foramen, is diagnosed in early childhood. In 95% percent of cases, it is accompanied by hydrocephalus and myelomeningocele. Types 3 and 4 are rare [1,2,6].

Impaired cerebrospinal fluid (CSF) flow is a common feature among all four types of ACM [2,6]. CSF fluid flow magnetic resonance imaging (MRI) is the gold standard for diagnosis, showing a disturbance in CSF dynamics. Symptomatic or worsening cases can be surgically corrected with suboccipital craniotomy, decompression, and duraplasty. Syringomyelia is associated with type 1 ACM in as many as 70–80% of cases [1] and may lead to more neurological symptoms.

There are several case reports of type 1 ACM in pregnancy, before and after surgical correction [7]. The main obstetric considerations are the effect of pregnancy on the progression of the disease and the management of labor and delivery. Uterine contractions, associated pain, and maternal effort of pushing during delivery are all thought to increase intracranial pressure (ICP), which can be potentially deleterious for someone with ACM [1].

A review of the literature suggests that most patients' symptoms tend to worsen during pregnancy. Reports from as far as back as 1970, limited as they may be, describe women with corrected and uncorrected ACM having vaginal deliveries and cesarean sections without complications [7].

The choice of anesthesia for labor analgesia or operative delivery in women with ACM continues to be an area of debate. Pregnancy and labor are associated with a rise in CSF pressure [8]. In ACM, there is a differential effect between the cranial CSF pressure above the foramen magnum and the spinal CSF pressure below.

Epidural anesthesia has been used in many cases without complications, although there is the theoretical risk of dural puncture worsening this differential effect and potentially tonsillar coning [9]. There is also some concern that the altered anatomy associated with ACM and its surgical correction may render epidurals ineffective.

Spinal anesthesia can theoretically cause compression of the structures at the level of the foramen magnum which will likely lead to raised ICP and its sequelae. There are reports of pregnant women with ACM having spinal anesthesia for cesarean section with and without complications. At least two cases detail women developing symptoms after epidural with or without spinal anesthesia with inadvertent dural puncture during epidurals being subsequently diagnosed with ACM [10,11].

GA has also been used in women with ACM without complications. The main concerns are of hypertension and raised ICP during induction, as well as head/neck extension during intubation causing further damage to the existing malformation. Some have advocated awake fiber-optic intubations [9].

The main obstetric considerations for disc prolapse in pregnancy include the management of pain and other symptoms, monitoring for the worsening of neurological sequelae, including cauda equina syndrome, deciding on the mode of delivery and positioning for vaginal delivery, as well as the choice of anesthesia for labor and delivery. Lumbar disc prolapse on its own does not affect pregnancy, though some women have had labor induced early due to worsening symptoms. Physical therapy options include reduced mobility, wearing flat shoes, bed rest with foot elevation, and exercises to increase core strength [12].

There are limited analgesic options in pregnancy, mainly restricted to paracetamol and opioids, which have the added risk of dependence and neonatal abstinence syndrome (NAS). Muscle relaxants are generally contraindicated in pregnancy, except for cyclobenzaprine, which is classed as FDA Category B [12]. In severe cases, epidural injection of steroids and nerve blocks have been employed. As decreased mobility due to progressive pain and radiculopathy can increase the risk of venous thromboembolism, patients should be risk assessed for thromboprophylaxis.

Patients with spinal canal pathology, like lumbar disc disease and spinal surgeries, are often not considered to be candidates for neuraxial blocks for fear of exacerbating the pre-existing neurological deficits or development of new neurological symptoms. There are reports of surgical decompression of severe lumbar disc prolapse in pregnancy, with laminectomy, discectomy, micro-discectomies all performed safely during pregnancy, preferably under epidural anesthesia [12,13].

Uncomplicated vaginal deliveries are widely reported in women with lumbar disc prolapse, before and after spinal surgery. Care should be taken with positioning during labor, with supine position with gentle leg abduction preferred. A theoretical concern exists that the lithotomy position can aggravate an existing disc prolapse and worsen symptoms.

## 4 Conclusion

This is a case of type 1 ACM and symptomatic lumbar disc prolapse successfully managed in pregnancy, with an uncomplicated emergency cesarean section under spinal anesthesia. As far as we are aware, this is the first reported case of both pathologies in one pregnant woman. This case highlights the importance of multidisciplinary collaboration in managing complex cases.

ACM in pregnancy is rare, and patient care should be individualized, with multidisciplinary involvement. Low back pain in pregnancy should be given serious consideration when patients present with severe radicular pain associated with neurological symptoms. These patients should have an urgent MRI and neurosurgical review. Spinal surgery can be safely performed at any stage of pregnancy, as required.
